# Quantifying Collective Performance in Rugby Union

**DOI:** 10.3389/fspor.2019.00044

**Published:** 2019-10-11

**Authors:** Guillaume Saulière, Jérôme Dedecker, Issa Moussa, Julien Schipman, Jean-François Toussaint, Adrien Sedeaud

**Affiliations:** ^1^IRMES, Institut de Recherche bio-Médicale et d'Epidémiologie du Sport, EA7329, INSEP & Université Paris Descartes, Sorbonne Paris Cité, Paris, France; ^2^Laboratoire MAP5, Université Paris Descartes, Sorbonne Paris Cité, Paris, France; ^3^Centre d'Investigations en Médecine du Sport, Hôtel-Dieu, Assistance Publique - Hôpitaux de Paris, Paris, France

**Keywords:** rugby, collective performance, collective efficacy, group dynamics, social networks

## Abstract

**Objectives:** The aim of this study was to quantify collective experience based on cumulative shared selections of players and to assess its impact on team performance in international rugby union. We assume that the greater the experience, the better the group will perform.

**Methods:** Scoresheets of all games involving at least one of all 10 nations participating at the Rugby Championship and the Six Nations Championship were collected from the end of the 1999 Rugby World Cup (RWC) up to the 2015 RWC. A single indicator quantifying the cumulative shared selections (CSS, the number of selections that each player has shared with the other ones) was computed for each match as a key collective experience indicator. The World Rugby Ranking points of each nation and the percentage of victories were used to estimate team performance. The study period was divided into sequences of 4 years corresponding to the period between two consecutive RWCs. For each sequence and nation, slopes and intercept of CSS trends were computed along with victory percentage and mean ranking points. Multiple linear regression analysis was used to establish the associations between team performance and experience.

**Results:** In regards to the CSS trends, both intra- and inter-nation variability appears to exist. Positive and negative slopes can be observed for the same team from one 4-year cycle to the next. Still, CSS slope is found to be significantly associated with both ranking points (*p* value = 0.042, *R*^2^ = 0.13) and victory percentage (*p* value = 0.001, *R*^2^ = 0.42).

**Conclusion:** The evolution of the CSS that quantifies the collective experience of a team is linked to its performance. Such an indicator could be helpful in the decision-making process of national coaching staff.

## Introduction

Many studies have shown the importance of investigating cohesion and collective efficacy factors as key performance indicators in team sports (Heuzé et al., 2006; Bourbousson et al., 2010; Marcos et al., 2010; Leo et al., 2013; Fransen et al., 2015; Sedeaud et al., 2017). In rugby union, no significant effort can be performed without the help and cooperation of every player. In this context of cooperation, a team's performance can be described both by its productivity and by the sum of its players' abilities. Hence, the main goal of a coaching staff is to enable the team's performance to be greater than the simple sum of the parts.

To do so, the coaching staff need to create cohesion and collective efficacy, the two have been proven determinants of performance. Qualitative aspects of collective experience on individual and collective performance have been explored. Heuzé et al. (2006) established that teammate's from more cohesive teams share stronger beliefs in their team. This enhances individuals' perception of collective efficacy thereby improving cohesion. In parallel, Marcos et al. (2010) added that improving team cohesion in basketball ameliorates a teammate's perception of efficacy. Moreover, Leo et al. (2013) stated that players with higher cohesion and collective efficacy profiles belonged to the best teams. Generating synergy through a coordinated effort which allows each member to maximize their strengths and minimize their weakness (Jain, 2009) is a long-term process (Sedeaud et al., 2017). It requires coordination based on shared knowledge and action (Eccles and Tenenbaum, 2004) that teammates can only achieve through time spent together and traditional methods of team-building (Shearer, 2015). Thus, impact of cohesion and collective efficacy gathered through collective experience has been widely qualitatively correlated with improvement of team performance.

Fewer studies have led to quantify player profiles and their potential relation on performance. Individual experience among the collective is a determinant for the team to perform. Previous studies have found that rugby union international players may be selected because of greater skill and experience (Walsh et al., 2007; Gabbett and Ryan, 2009; Hendricks and Lambert, 2014). Furthermore, the sum of individual experience among the squad has also been reviewed. Sedeaud et al. (2012) showed that teams which include forwards with previous World Cup experience perform better. Moreover, specific analysis on collective effectiveness of French national rugby team showed that more experienced forwards surround the best halfbacks, locks and centers (Sedeaud et al., 2017). Impact of player turnover rate on results has also been analyzed in rugby union and football. A high rate of turnover in the French national rugby team between two consecutive games is associated with the loss of the game (Sedeaud et al., 2017). Similar results have been found in football by Carling et al. (2015). The best results of one football team over 5 seasons were obtained when the fewest number of players were alternated over the season. This team won the national championship with the same 10 players involved in at least 75% of the total minutes played during the season. During the other seasons, <6 players had this characteristic of high play-time. This was potentially due to higher player availability and low injury incidence, which had a significant influence on team success (Hägglund et al., 2013). Those results imply that squad management strategies directly foster team performance.

The impact of team experience on team performance has been analyzed through qualitative estimators such as cohesion and collective efficacy, and then quantified by indicators summing individual experiences or assessing a global estimation of the group experience. The purpose of this study was to quantify team experience and its evolution through time, based on cumulative shared selections of players. Then, to assess its impact on team performance. As specified by Shearer (2015), the prestige and the 4-year cycle between two Rugby World Cup events make this competition an ideal field of investigation. As shown in a recent paper from Mukherjee et al. (2019), we assume that a strong link exists between a team's history and its performance: that the greater the experience, the better the group's performance will be.

## Methods

### Data Collection

After obtaining the approval of the National Institute of Sport, Expertise, and Performance ethics committee (*Conseil Scientifique Medical et de Formation, CSMF*), scoresheets of all games involving at least one of all 10 nations participating at the Rugby Championship (*Argentina, Australia, New Zealand, and South Africa*) and the Six Nations Championship (*England, France, Ireland, Italy, Scotland, and Wales*) were collected from the end of the 1999 Rugby World Cup (RWC) up to the 2015 RWC final. Date of the game, competition, points scored and conceded, names and positions (forwards and backs) of players involved as starters were recorded for each international game. Data were collected from an up-to-date rugby news and statistics website: www.statbunker.com. The site is updated after every game, and has a comprehensive list of statistics, covering all aspects of rugby union. Additional to the scoresheet data, World Rugby Ranking points of each nation were recorded weekly from its introduction at the beginning of 2004 up to the last game covered by our study period. Ranking points were collected on the World Rugby website: www.worldrugby.org.

### Team Experience Indicator: Cumulative Shared Selections

First, we quantified team experience at a given game. A single indicator based on the set-lists of the starters selected by the head coach was produced. For each game, number of selections that each player has shared with the others during the previous games were computed and added up as follow.

Let's define for each national team a network with *n* vertex corresponding to every players selected over *m* matches through a certain period of time. Different vertex can or cannot be linked by edges *E* depending on whether the players had played together at least once. Edges are weighted according to the number of match shared by the players: if two players *i and j* (*i and j* ∈ {1, …, *n*}) play at a given match *k*, then ${E^{k}}_{i,j}=1$. If they don't or only one does play, then ${E^{k}}_{i,j}=\;0$.

Hence, the number of CSS of a team at a given game *g* (*g* ∈ {1, …, *m*}) is

$$\begin{array}{l} CSS_{g}=\frac{1}{2}\times \overset{g-1}{\underset{k=1}{\sum }}\left(\overset{n}{\underset{i=1}{\sum }}\left(\overset{n}{\underset{j=1,j\neq i}{\sum }}\left(E_{i,j}^{k}\right)\right)\right). \end{array}$$

This calculation quantifies the Cumulative Shared Selections (CSS) of a team at a given game. The number of CSS was computed over the entire period (1999–2015). Then we aimed to analyze the evolution of the team experience indicator through time. As implied by Shearer (2015), we chose to divide our study period into sequences of 4 years, each sequence corresponds to the period between two consecutive RWCs. Mean number of CSS was computed by nation for each sequence.

### Slope, Intercept, Ranking Points, and Victory

The behavior of the team experience indicator through time was analyzed. Slopes and intercepts from linear regression between CSS's evolution and games played were computed and compared for each sequence and nation. The slope between two RWCs reflects how the CSS evolved in preparation for the following RWC. The intercept is used to for analysis adjustment over the basal team experience at the beginning of a RWC cycle.

Both World Rugby Ranking points and percentage of victories were used to estimate national team performance. From the same perspective of analyzing evolution of the team experience, mean ranking points and victory percentage were computed by nation for each 4 year time block.

### Statistical Analysis

Data are reported as mean ± standard deviation. Multiple linear regression analysis (see Table 1) is used for establishing the potential associations between team performance and team experience indicators which are entered as dependent variable. Please see below the multiple regression models:

$$\begin{array}{l} \;ranking\;points=\beta_{0}+\beta_{1}\times CSS\;slope\\ \;+\beta_{2}\times CSS\;intercept+\varepsilon \\ victory\;percentage=\beta_{0^{\prime }}+\beta_{3}\times CSS\;slope\\ \;+\beta_{4}\times CSS\;intercept+\varepsilon^{\prime } \end{array}$$

Results are considered significant at *p* < 0.05. All statistical analyses were performed using R (version 3.3.2; The R Foundation for Statistical Computing, Vienna, Austria).

**Table 1:** Multiple linear regressions results.

	**Coefficient (β)**	**SE**	**95% CI**	***p*-value**
**Ranking points**				
CSS slope	0.36	0.16	0.074–0.643	0.042
CSS intercept	0.005	0.005	−0.003–0.013	0.330
**Victory percentage**				
CSS slope	1.78	0.45	0.992–2.568	0.001
CSS intercept	0.02	0.013	−0.005–0.04	0.193

*Effects of team experience indicators over team performance*.

Team experience estimated by the number of CSS computed in the first years of our study period is biased and does not take into account all previous games shared by the players. Therefore, specific analysis is conducted over three 4-year sequences between 2004 and 2015.

## Results

### Variable Descriptions

The study period covered three sequences of 4 years between the 2003, 2007, 2011, and 2015 RWCs. Mean number of games played by the 10 nations over a sequence is 45.9 ± 6.9, involving an average of 62.1 ± 7.6 different players. Regarding the experience indicators, mean number of CSS over a sequence is 860.4 ± 284.9 and it increases, on average, with a slope of 7.9 ± 8.1 and an intercept of 677.0 ± 287.5. Regarding performance indicators, the 10 nations completed the sequences with mean ranking points of 82.21 ± 5.59 and a mean victory percentage of 55.27 ± 18.95.

**Figure 1:**
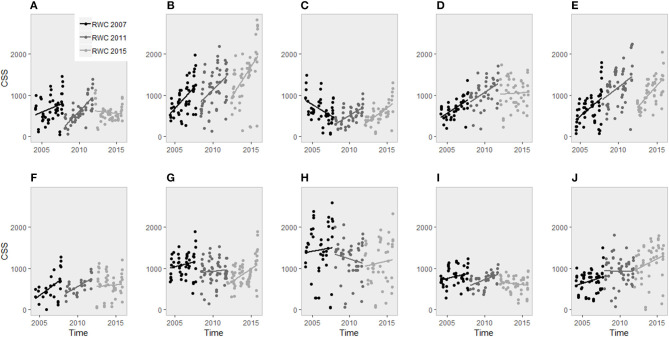
CSS evolutions over the 2007, 2011, and 2015 RWC. **(A)** France, **(B)** New Zealand, **(C)** England, **(D)** Italy, **(E)** South Africa, **(F)** Argentina, **(G)** Australia, **(H)** Ireland, **(I)** Scotland, and **(J)** Wales.

Figure 1 illustrates CSS evolutions over the 2007, 2011, and 2015 RWC for the 10 teams involved in four and six nations. Table 2 displays experience and performance indicators by nations for each sequence. Looking at England's trend of CSS, the slope leading to the first world cup is −9.03 resulting in a 2nd place ranking. Then, the slopes of the last two RWC period are 10.6 and 12.65 leading to a defeat in the quarter final and not progressing past the group phase, respectively. In the meantime, those slopes increase through the 3 periods and are associated with a 10% increase in victories per 4-year sequence (from 44 to 64%). In 2007, the RWC winner displayed a slope of 12.99 (Table 2). During the entire 12 years of follow-up, only Wales and New Zealand share a continuous progression of their CSS (Figure 1) and an uninterrupted improvement of victory percentage and ranking score (Table 2).

**Table 2:** Descriptions of the variables from the 10 nations over the 2007, 2011, and 2015 RWC.

**Nation**	**RWC**	**CSS**	**CSS slope**	**CSS intercept**	**Ranking points**	**Victory percentage**	**RWC result**
New Zealand	2007	911.17 ± 403.48	16.59 [9.61; 23.60]	504.52 [307.71; 701.32]	92.59 ± 1.50	87.5	Quarter finalist
	2011	1,147.60 ± 488.61	14.39 [6.98; 21.80]	744.67 [506.22; 983.13]	91.39 ± 1.49	83.64	Winner
	2015	1451.26 ± 632.65	21.27 [11.43; 31.11]	876.95 [571.53; 1182.36]	92.70 ± 1.06	94.12	Winner
France	2007	675.49 ± 324.91	7.21 [0.88; 13.54]	495.33 [313.55; 677.11]	85.18 ± 1.26	70.83	Semi finalist
	2011	627.04 ± 304.52	18.20 [13.78; 22.62]	208.42 [91.74; 325.10]	82.02 ± 1.91	60,00	Finalist
	2015	577.60 ± 206.58	−1.47 [−6.29; 3.35]	611.42 [484.21; 738.62]	81.58 ± 1.66	46.51	Quarter finalist
England	2007	645.06 ± 309.81	−9.03 [−15.25; −2.81]	861.73 [690.28; 1033.18]	83.39 ± 4.78	44.68	Finalist
	2011	515.53 ± 212.82	10.6 [6.83; 14.37]	271.83 [172.25; 371.42]	82.33 ± 1.5	54.55	Quarter finalist
	2015	561.18 ± 259.9	12.65 [7.97; 17.33]	270.23 [146.62; 393.84]	84.24 ± 1.57	63.64	Other
Italy	2007	679.85 ± 238.3	11.55 [6.3; 16.79]	437.35 [310.9; 563.81]	72.81 ± 1.44	30,00	Other
	2011	1,061.93 ± 328.79	11.72 [4.01; 19.42]	810.04 [619.93; 1000.16]	73 ± 1.08	21.43	Other
	2015	1,045.64 ± 358.9	1.07 [−7.33; 9.47]	1,021.04 [799.23; 1242.84]	73.31 ± 2.01	24.44	Other
South Africa	2007	684.96 ± 386.84	12.99 [6.97; 19.01]	334.24 [147.4; 521.08]	85.74 ± 1.97	67.31	Winner
	2011	1,194.94 ± 483.66	12.72 [3.52; 21.92]	876.93 [612.55; 1141.32]	88.54 ± 2.07	63.27	Quarter finalist
	2015	1,060.6 ± 356.13	16.97 [11.34; 22.59]	644.94 [486.61; 803.27]	86.91 ± 2.17	69.57	Semi finalist
Argentina	2007	543.71 ± 331.87	24.1 [11.06; 37.14]	194.28 [−22.22; 410.78]	79.29 ± 3.53	64.29	Semi finalist
	2011	575.88 ± 196.92	18.34 [9.07; 27.6]	346.66 [214.24; 479.08]	81.01 ± 2.83	37.5	Quarter finalist
	2015	585 ± 319.17	2.44 [−4.51; 9.39]	526.34 [334.73; 717.95]	77.72 ± 2.58	28.26	Semi finalist
Australia	2007	1,085.35 ± 293.76	4.41 [−1.48; 10.3]	975.04 [805.79; 1,144.28]	86.53 ± 1.48	60.42	Quarter finalist
	2011	927.15 ± 296.89	2.22 [−2.85; 7.29]	864.91 [701.76; 1028.06]	85.86 ± 1.17	59.26	Semi finalist
	2015	874.39 ± 320.51	9.13 [4.53; 13.73]	609.64 [456.34; 762.95]	86.28 ± 1.81	60,00	Finalist
Ireland	2007	1,441.59 ± 646.01	4.49 [−11.11; 20.09]	1,340.61 [937.62; 1743.61]	82.27 ± 1.56	61.36	Other
	2011	1,217.73 ± 457.29	−4.16 [−14.79; 6.47]	1,313.41 [1032.61; 1594.22]	81.01 ± 2.3	56.82	Quarter finalist
	2015	1,133.61 ± 543.61	5.74 [−7.31; 18.8]	1,004.39 [667.06; 1341.71]	82.08 ± 2.75	61.9	Quarter finalist
Scotland	2007	796.95 ± 231.49	5.43 [−0.35; 11.2]	680.22 [537.69; 822.76]	75.34 ± 1.31	33.33	Quarter finalist
	2011	724.92 ± 171.38	8.33 [4.16; 12.5]	558.28 [462.65; 653.92]	76.98 ± 1.87	42.11	Other
	2015	624.79 ± 186.72	−1.76 [−5.68; 2.17]	667.83 [557.39; 778.26]	76.49 ± 1.22	37.5	Quarter finalist
Wales	2007	693.21 ± 269.52	5.35 [−0.14; 10.84]	562.16 [407.68; 716.63]	78.2 ± 2.48	45.65	Other
	2011	931.72 ± 332.22	0.33 [−6.29; 6.94]	923.35 [729.58; 1117.12]	79.43 ± 1.89	51.02	Semi finalist
	2015	1,135.6 ± 439.76	8.76 [−0.19; 17.71]	921.03 [669.05; 1,173]	81.65 ± 2.17	54.17	Quarter finalist

### Multiple Linear Regressions

In multiple linear regressions, CSS slope is found to be significantly associated with both ranking points (*p* value = 0.042, *R*^2^ = 0.13) and victory percentage (*p* value = 0.001, *R*^2^ = 0.42), adjusted on the CSS intercept (Table 1).

## Discussion

This study is the first to create a collective performance indicator and reveal its association with ranking and victory.

We have investigated the 4-year cumulative shared selections evolution of the national rugby teams involved in four and six nations between consecutive RWCs from 2004 to 2015. A general indicator quantifying collective experience was computed. Its impact on rugby union game results was assessed. Previous work introduced the idea that shared selections are highly involved in rugby union teams' performance and that time is needed to let collective effectiveness emerge (Sedeaud et al., 2017). Sedeaud et al. concentrated their investigations on shared experiences between pairs of players (halfbacks, locks, or centers). Investigating shared experiences between all players reveals a new way to test these relationships.

Team cohesion and collective efficacy are widely used to qualify collective experience (Heuzé et al., 2006; Bourbousson et al., 2010). These qualities are acquired through the time players spent with each other (Shearer, 2015); CSS is proposed as a global statistical tool to quantify all the experiences shared between the players during successive games. By definition, this indicator changes after each game, making it a natural candidate to analyze team dynamic construction over a defined period. Slopes and intercepts of CSS evolution over 4-year cycles preceding RWCs put into numbers the strategies established by the coaching staff to create their teams.

### CSS Trends and Performance

With regards to the CSS trends, its slopes and intercepts, both intra- and inter-nation variability appear to be wide. Positive and negative slopes can be observed for the same team from one 4-year cycle to the next. For example, England presents opposite CSS evolutions leading up to the 2007, 2011, and 2015 RWCs with a negative slope followed by two positive ones. Those slopes are associated with a 10 percent increase in victories per 4-year sequence (from 44 to 64%). On the other hand, South Africa seems to apply a different strategy for two successive RWCs, with two consecutive positive slopes (increasing team building), with the second one starting precisely where the first one ends, implying some continuity in the policy of player selection. Such a continuity observed for the 2011 RWC 4-year cycle could be explained by the confidence in the players after their success in the 2007 RWC. Nonetheless, a continuous improvement in CSS during a long period is paradoxical. It implies a general aging of the group which in reality is naturally topped out by the physical demands. Mean ages of RWC players of 25 years for backs and 27 for forwards (Sedeaud et al., 2012) reflect such a reality. The construction of a collective workforce that shares consequent playing time also faces the necessary youth physical prerequisites. Indeed, specific positions in rugby union demand repeated sprints, changes of direction, and capacities that generally decline with age (Berthelot et al., 2012; Marck et al., 2017).

Concerning multiple linear results exploring effects of experience indicators over performance ones, CSS slope is found to significantly impact both ranking points (*R*^2^ = 0.13) and victory percentage (*R*^2^ = 0.42) while CSS intercept does not. Despite the fact that a statistical relation between CSS slopes and team performance is highlighted, results must be interpreted cautiously. Only 13 and 42% of the observed variations can be explained by the model's inputs for ranking points and victory percentage, respectively. This remains an interesting result, which may imply that improving CSS (i.e., international games played together) over the entire 4-year period prior to the RWC is more important that initial collective experience garnered in the past. In other words, progression is a key variable rather than the starting point. These results are consistent with previous conclusions showing that the same halfbacks, locks, or centers selected over time, obtained at the end of their common career, a winning percentage similar to the team's average (Sedeaud et al., 2017).

### CSS and Managing Strategies

The rendez-vous of the RWC every 4 year allows to analyze how teams are built while preparing for such competitions (Shearer, 2015). For the best team, optimizing shared selections is a suitable strategy, but one that takes time and should be considered to have some limits. Furthermore, high intercepts are more often associated with negative or slight slopes: Australia, England in 2007, Ireland during the entire 12 years, and Italy in 2015. This reveals that the first year after an RCW may be crucial, that early choices of head coaches may be a primary determinant for the rest of the 4-year period (e.g., keeping attention to the incorporation process, mixing experienced players with young ones). New Zealand parallels these trends with a slight increase of intercepts, which results in a mix between players. This mix brings together experienced players, indispensable to the tactical group cohesion and optimal operation and progressively incorporates young players at the peak of their physical performance.

### Perspectives and Limitations

The aim of this study was to quantify time spent together by the players and test whether a strong collective experience was correlated with better collective performance. Taking into account the fact that a squad can be composed of some players who play for the same club might be relevant. Groll and Abedieh (2013) showed in football that too many players coming from the same clubs negatively impacts the national selection's performance because of a lack of diversity (too many players scattered in too few clubs). In addition, it leads to a lack of knowledge of foreign game features. Future studies must investigate the impact of this factor in rugby union for comparison with results found by Groll in football. Comparisons between TOP 14 French players who come from many different clubs, with the New Zealand players involved in Super Rugby playing primarily for only 5 franchises could be relevant in order to identify different efficiency collective patterns.

By their construction and definition, team experience and performance indicators used in this study are macroscopic and unfortunately led to a loss of information (e.g., the victory percentage give no clue on the quality of games results). As a consequence of this limitation, some examples from our data do not necessarily support linear regression results. One can cite the negative CSS slope of England in 2007 leading them to an honorable 2nd place, or the strong slope (11.72) and intercept (810.04) of Italy associated to their lowest victory percentage (21.43). More accurate performance indicators must be taken in consideration in future analysis. Regarding team experience indicators, social network analysis (SNA) might help to provide deeper investigations. SNA techniques are increasingly applied to analyse behaviors within teams and inter-player interactions during games (Grund, 2012; Araújo and Davids, 2016; Cintia et al., 2016; Pina et al., 2017; Ribeiro et al., 2017). Bipartite structure of complex networks as described in Guillaume and Latapy (2004) might be an interesting tool to deeply scope the evolution of players' interactions through time and successive games, and might reveal individual or specific positions influencing the entire team evolution.

Due to a lack of information, we were not able to account for the impact of injuries on CSS evolution. The negative effect on team performance due to players unavailability caused by injuries has been shown in football (Parry and Drust, 2006; Hägglund et al., 2013; Carling et al., 2015; Windt et al., 2018). As reported by Bengtsson et al. (2013), the influence of team rotation strategies on (a) team performance and (b) injury rates remains unclear. While our results shed light on the first relationship, further research must help to define the second one. Studying CSS impact on a team performance during a championship with large injury incidences (such as in rugby union) might help to better understand inherent relations between squad management, team performance and player injury.

## Conclusion

For the first time, a single estimator (CSS) allows us to relate the evolution of a team's experience and its performance through time. This study reveals the potential of this indicator. It would be captivating to transpose this methodology to other team sports and other competition formats, such as a championship. Investigating more precisely the links between players through social network analyses would also make it possible to discretize relationships and detect key individuals or groups of individuals.

National coaching staff need to create the most competitive squad for each competition. They have to decide what to do with the time and few games to play that are given to them. The number of cumulative shared selections is a parameter that could help them in the decision-making process.

## Data Availability Statement

The datasets generated for this study are available on request to the corresponding author.

## Author Contributions

GS, JD, IM, JS, J-FT, and AS conceived, designed, performed, and analyzed the research. GS and AS wrote the manuscript. All authors read and approved the final manuscript.

### Conflict of Interest

The authors declare that the research was conducted in the absence of any commercial or financial relationships that could be construed as a potential conflict of interest.
